# Dogs and wolves do not differ in their inhibitory control abilities in a non-social test battery

**DOI:** 10.1007/s10071-018-1216-9

**Published:** 2018-10-03

**Authors:** Désirée Brucks, Sarah Marshall-Pescini, Friederike Range

**Affiliations:** 1 Domestication Lab, Wolf Science Center, Konrad Lorenz Institute of Ethology, University of Veterinary Medicine, Vienna, Savoyenstraße 1a, 1160 Vienna, Austria; 2 Comparative Cognition, Messerli Research Institute, University of Veterinary Medicine Vienna, Veterinärplatz 1, 1210 Vienna, Austria

**Keywords:** Inhibitory control, Domestication, Dogs, Wolves, Test battery

## Abstract

Being able to inhibit certain behaviours is of clear advantage in various situations. In particular, it has been suggested that inhibitory control plays a role in problem-solving and cooperation. Interspecific differences in the capacity for inhibitory control have been attributed to social and ecological factors, while one additional factor, namely domestication, has received only little attention so far. Dogs are an interesting species to test the effects of socio-ecological factors and also the influence of domestication on inhibitory control abilities. While dogs might have been selected for enhanced inhibition skills during domestication, the predictions derived from their socio-ecological background are reversed. Wolves are cooperative hunters and breeders, while dogs predominately scavenge and raise their young alone, accordingly, it would be predicted that dogs show impaired inhibitory control abilities since they no longer rely on these coordinated actions. To test these hypotheses, we assessed inhibitory control abilities in dogs and wolves raised and kept under similar conditions. Moreover, considering the problem of context-specificity in inhibitory control measures, we employed a multiple-test-approach. In line with previous studies, we found that the single inhibition tests did not correlate with each other. Using an exploratory approach, we found three components that explained the variation of behaviours across tests: motivation, flexibility, and perseveration. Interestingly, these inhibition components did not differ between dogs and wolves, which contradicts the predictions based on their socio-ecological backgrounds but also suggests that at least in tasks with minimal human influence, domestication did not affect dogs’ inhibitory control abilities, thus raising questions in regard to the selection processes that might have affected inhibitory control abilities during the course of domestication.

**Electronic supplementary material:**

The online version of this article (10.1007/s10071-018-1216-9) contains supplementary material, which is available to authorized users.

## Introduction

The ability to refrain from exhibiting immediate, potentially disadvantageous, behaviours in inappropriate situations certainly provides benefits. Indeed, inhibitory control seems to be involved in several behaviours such as problem-solving (e.g. Müller et al. 2016; Vlamings et al. 2010), behavioural flexibility (Amici et al. 2008; Santos et al. 1999), and has been used as a proxy for cognition (MacLean et al. 2014) and general intelligence (Beran and Hopkins 2018), hence highlighting the importance of understanding and assessing this ability. Importantly, inhibitory control is not a unitary construct but rather context-specific (e.g. Tsukayama et al. 2012) and individual performances seem to differ between inhibitory control tasks (e.g. Amici et al. 2008; Addessi et al. 2013; van den Bergh et al. 2006), Accordingly, different classifications for inhibitory control have been put forward (i.e. cognitive inhibition = regulate low-level actions not relevant to the task, motor inhibition = inhibition of prepotent responses, self-control = choice for a delayed reinforcer; see Beran 2015 for a review). Recently the validity of this distinction was directly assessed in dogs by utilizing a multiple tests approach (Bray et al. 2013; Brucks et al. 2017a). These studies revealed that tests aimed at measuring the same aspects of inhibitory control (e.g. motor inhibition) are not correlated with each other, but rather seem to be composed of different inhibition components (e.g. persistency, compulsivity, decision speed in Brucks et al. 2017a).

Differences in inhibitory control abilities occur not only at the individual but also at the species level (e.g. Amici et al. 2008; Lakshminaryanan and Santos 2009; Stevens et al. 2005), leading to different hypotheses regarding the potential causes for this variation. A number of hypotheses relate to the complexity of a species’ social organization, as a potentially determining factor (e.g. Amici et al. 2008; Aureli et al. 2008). It has been argued that living in complex social organizations, such as fission–fusion groups, requires, apart from enhanced social cognitive abilities (e.g. individual recognition, memory of hierarchy, etc.), also inhibitory control and behavioural flexibility in interacting with changing social partners (Aureli et al. 2008). And indeed, one study found that among primate species those living in social groups with higher levels of fission–fusion dynamics exhibited better inhibitory control abilities (Amici et al. 2008). Another social factor that potentially requires enhanced inhibitory control abilities is cooperative interactions. Engaging in coordinated behaviours requires individuals to inhibit immediate actions and instead take the partner(s)’ behaviour into account. For example, cooperatively breeding species need to coordinate their actions (i.e. vigilance, group and territory defence, and care-giving), to successfully rear their offspring (e.g. Burkart and van Schaik 2010). But also during cooperative hunting, animals need to inhibit the urge to attack the prey by themselves and instead monitor and coordinate their action with that of the other group members (Bailey et al. 2012; MacNulty et al. 2007). Moreover, recent findings show that inhibitory control is also involved in the expression of inequity aversion, a behavioural mechanism thought to stabilize cooperation in the long-term (Brucks et al. 2017b).

Alternatively, it has been hypothesized that differences in inhibitory control abilities are related to the species’ feeding ecology. Accordingly, species with longer food processing times, such as gumnivorous species, may have evolved better inhibitory control abilities than species, which have immediate access to food such as frugivorous or insectivorous species (Stevens et al. 2005). In addition, the availability of resources might have shaped a species’ inhibitory control abilities with reliance on small and unpredictable food resources requiring better inhibition skills (Rosati et al. 2007).

Even though dogs and wolves share a common ancestor, they are adapted to different ecological niches during domestication and their social organization has diverged drastically (see Marshall-Pescini et al. 2017a for a review). Wolves engage in-group hunting to take down large prey (Mech et al. 2015). Conversely, while free-ranging dogs, which make up 83% of the world dog population (Lord et al. 2013), have been observed to occasionally hunt wildlife (i.e. dingos; Vanak and Gompper 2009) they generally rely more on a scavenging lifestyle. Specifically, dogs have adapted to live in proximity to human settlements and depend on human waste (Sen Majumder et al. 2016), which is a rather predictable and easily accessible food source. This change in the ecological niche has likely also affected dogs’ social organization. Finding human waste does not require much coordination or cooperation among pack members but instead, dogs forage individually (Sen Majumder et al. 2014). Pups start foraging at a young age and consequently do not need to be provisioned by adults and indeed, free-ranging dogs do not show the same stable family packs common in wolves, but rather appear to form multi-male multi-female groups with females rearing their pups mostly alone (Lord et al. 2013; but see Pal 2005 where some limited paternal care has been observed). Wolves, on the contrary, live in family packs consisting of a breeding pair and their offspring, which cooperatively raise their young, by providing food for them during the first year of life (Mech and Boitani 2003). Indeed, also experimentally this difference in dogs’ and wolves’ cooperative abilities has been demonstrated recently (Marshall-Pescini et al. 2017b). While wolves were able to coordinate with their partners to gain access to food rewards in a loose-string paradigm, the dogs failed to take the partner’s behaviour into account and tried to access the rewards alone. Following the argumentation outlined above it would be expected that dogs exhibit less inhibitory control abilities than wolves considering their social organization (i.e. scavenging and maternal care in dogs vs. cooperative hunting and breeding in wolves) and ecology (i.e. stable resources vs. unstable resources).

Another aspect to consider aside from dogs’ socio-ecology, is the potential effect of human selection for specific traits during domestication on specific mechanisms such as inhibitory control. The domestication of dogs started thousands of years ago and resulted in a uniquely close relationship with humans (Kaminski and Marshall-Pescini 2014; Thalmann et al. 2013). The emotional reactivity hypothesis proposes that dogs were selected for a tamer and less aggressive temperament, which allowed them to apply their intraspecific behavioural repertoire also to interactions with humans (Hare et al. 2005; Hare and Tomasello 2005). An extension of this hypothesis was proposed later suggesting that the domestication of dogs started with a process of ‘self-domestication’, in which less fearful and less aggressive wolves experienced a selective advantage and could exploit human settlements before in a second step humans started to selectively breed dogs (Hare et al. 2012). Such a selection on systems mediating fear and aggression might have affected their inhibitory control abilities as well. For example, in rats more aggressive individuals also show inhibition problems and cannot tolerate delayed rewards (Van den Bergh et al. 2006). Likewise, impulsive dogs often develop behavioural problems, such as impulsive aggression (e.g. Fatjó et al. 2005), which can additionally be triggered by fear (Archer 1976). Accordingly, animals that could inhibit their fear and/or aggression response might have had an advantage when interacting with humans. The synergistic hypothesis, suggests that dogs were directly selected for better inhibitory control abilities, which in turn increased their propensity to successfully cooperate with humans (Gácsi et al. 2009). Accordingly, if humans selected dogs based on their temperament (i.e. inhibition of fear and aggression; emotional reactivity/self-domestication hypotheses) or directly for their inhibition abilities (synergistic hypothesis), dogs should have derived generally enhanced inhibitory control abilities during domestication.

While dogs’ inhibitory control abilities have been tested in many different tasks (e.g. Bray et al. 2013; Brucks et al. 2017a; Leonardi et al. 2012; Müller et al. 2016) only recently wolves’ capacity for inhibitory control has been investigated directly (Marshall-Pescini et al. 2015). Initial studies proposed that dogs possess better inhibitory control abilities than wolves due to the fact that they were calmer (i.e. less struggling and biting) during handling and had less difficulty focusing on the human during testing (Gácsi et al. 2004, 2009). However, these studies were not specifically designed to test inhibitory control but rather derived these assumptions based on behavioural variables (i.e. struggling during handling, aggression towards experimenter, gaze) obtained during human-communicative pointing tasks (Gácsi et al. 2009) and physical problem-solving tasks (Udell 2015). In particular, the fact that wolves did not like being contained by humans might have nothing to do with their inhibition capacities but rather shows their low frustration tolerance since they were hindered to access the food rewards in front of them. Frustration is operationally defined as an aversive emotional reaction to incentive omission (Amsel 1992). Applied to this context, wolves might struggle because they want to get access to food and get frustrated because they are restraint; however, these results can also be explained with a lack of inhibitory control (i.e. wolves struggle because they cannot inhibit their urge to go to the food). These two explanations are not necessarily mutually exclusive, since having a certain frustration tolerance could be linked to enhanced inhibitory control as well. One recent study, however, tested dogs’ and wolves’ inhibitory control abilities directly by presenting them two classic inhibition tasks (Marshall-Pescini et al. 2015). In the cylinder task, the animals were trained to retrieve a reward out of an opaque cylinder, which was open on both ends. After several training trials, the cylinder was made transparent; hence the animals could see the reward inside. Accordingly, to get access to the reward they needed to inhibit reaching for the reward directly (which would result in bumping into the transparent surface) but instead go to the sides of the cylinder and access the reward from there. The other inhibition test was a detour task, in which the animals were required to walk away from the food reward and first make a detour around a V-shaped fence to reach a reward. While the dogs outperformed the wolves in the cylinder task, the exact opposite pattern was observed in the detour task, hence rendering a conclusion about dog-wolf differences difficult.

Considering these studies, no conclusions regarding the differences in dogs’ and wolves’ inhibitory control abilities could be drawn. Furthermore, one aspect that was not taken into account in the aforementioned comparative studies, is the fact that inhibitory control has often been shown to be context-specific (Bray et al. 2013; Brucks et al. 2017a; Tsukayama et al. 2012). Accordingly, it is possible that these studies assessed different aspects of dogs’ and wolves’ inhibition abilities. Consequently, to assess dogs’ and wolves’ overall inhibitory control capacities it is essential to apply a multiple-test approach.

To further evaluate the potential effects of domestication on inhibitory control abilities, we tested dogs and wolves raised and kept under the same conditions. We used a multiple-tests approach following the procedure of Brucks et al. (2017a) and incorporated tests, which aimed at measuring different aspects of inhibitory control (i.e. motor and cognitive inhibition). More specifically, we tested the animals motor inhibition skills in a detour-reaching test (‘box test’), and a ‘middle cup test’, whereas cognitive inhibition was assessed in a reversal-learning test. In addition, we implemented a test called the ‘buzzer test’, which measures motor inhibition while also entailing aspects of cognitive inhibition. All tests were conducted with as little human influence as possible to control for potential dog-wolf differences in social inhibition relating to the experimenter being present (e.g. Udell 2015). If the socio-ecological background is the main driver for sub-species differences in inhibitory control we would predict, that compared to equivalently reared wolves, dogs should show the following differences: decreased motor inhibition skills as measured in persistence in the box, buzzer and middle cup test, decreased cognitive inhibition on the buzzer and reversal-learning test, as well as higher levels of compulsive behaviour as demonstrated by repeated choices in the middle cup and reversal-learning test, and longer latency to response on the middle cup and reversal-learning test. Specifically, if we find the same inhibition components as in our previous study (Brucks et al. 2017a), dogs should be less persistent considering that scavenging requires less persistency than hunting (i.e. finding prey, stalking, coordination with other pack members). Moreover, dogs should be more compulsive than wolves, since making the same mistakes repeatedly would be devastating in an unstable environment (e.g. repeatedly chasing prey too early would lead to starvation). Furthermore, we predict that dogs exhibit a lower decision speed than wolves (e.g. dogs having more time to decide between places in which they can scavenge vs. wolves making quick decisions in the hunting context).

Conversely, if human-related selection for certain traits during domestication is responsible for differences in inhibitory control we would predict that dogs should show the following differences: increased motor inhibition skills as measured in the box, buzzer and middle cup test, increased cognitive inhibition in the buzzer and reversal-learning test, lower levels of compulsive behaviours and slower decision speed as assessed in the middle cup and reversal-learning test. These predictions are based on the assumption that humans have selected dogs for a calmer and more inhibited temperament (i.e. for dogs not trying the same behaviours repeatedly and inhibiting persistent fear/aggressive response).

## Methods

### Subjects

We tested 17 mixed-breed dogs (7F/10M, age: 4.43 ± 1.59 years.) and 12 timber wolves (4F/8M, 6.56 ± 1.68 years; see Supplementary Material Table S1 for individual characteristics). Dogs and wolves were raised and kept under similar conditions at the Wolf Science Center (WSC, http://www.wolfscience.at), Ernstbrunn, Austria. All tests were conducted between October 2016 and March 2017 in an indoor test room familiar to the animals. Testing was approved by the ethical committee of the University of Veterinary Medicine Vienna (approval number: 10/12/97/2013).

## Inhibition tests

Dogs and wolves were tested in four inhibitory control tests identical to those in Brucks et al. (2017a) but with apparatuses of enlarged dimensions. This test battery included two tests thought to measure motor inhibition (box and middle cup test), one test measuring cognitive inhibition (reversal learning test), and one test involving motor inhibition but also aspects of cognitive inhibition (buzzer test). Animals were tested individually and only one test was conducted per week, with the order of tests randomized and counterbalanced across subjects. All tests were conducted by experimenters familiar to the individuals.

### Box test

**Fig. 1:**
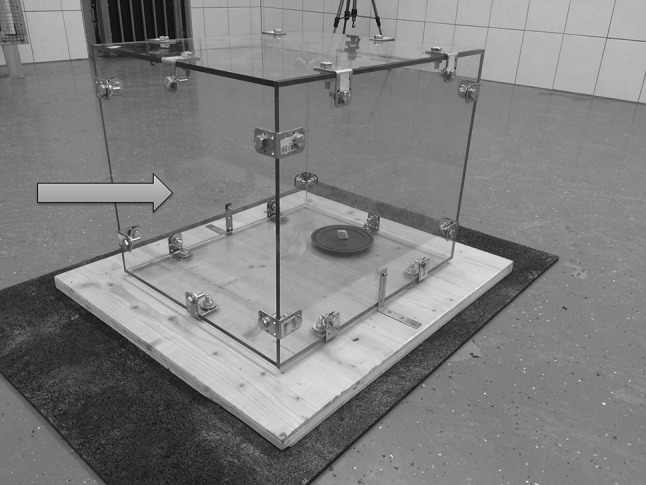
Transparent box used for the box test. The reward was placed on the blue plastic lid in the deep reward location. The blue arrow indicates the open side of the box

A piece of sausage was placed inside of a rectangular-shaped Plexiglas box (55.0 × 45.1 × 46.6 cm). One of the smaller sides of the rectangle was left out, hence allowing the animals to put their head inside the box. The box was screwed on a wooden board to enhance stability, and to prevent movements of the box it was placed on a rubber mat (see Fig. 1). To enhance the visibility of the rewards (i.e. pieces of sausage), they were placed on a plastic lid.

This test aimed at measuring the animals’ ability to inhibit the motor action of reaching for the reward directly through the transparent barrier. Additionally, a small cognitive component is involved since the animals need to navigate and find the open side of the box.

#### Procedure

Two pre-training trials were conducted, in which a piece of sausage was visibly placed on a plastic lid before putting the lid on the ground, once on either side of the covered box (2 m away from the individual). The animal was released from a starting position 2 m away from the fence and allowed to eat the reward. These pre-training trials were conducted to facilitate the association between lid and reward prior to testing.

Following this, the training phase began, in which the box remained covered (cardboard attached to walls of box), however, the animal witnessed how the experimenter placed the lid (with a reward) inside of the box. In doing so, the experimenter first caught the animal’s attention by calling its name, then visibly placed the reward on the lid, and then bend down to place the lid inside the box. The experimenter stepped back from the box (1.5 m) and looked to the floor. This was signal for the trainer to release the animal from the starting position (2 m away from box). After the animal had found and eaten the reward, it was called back to the starting position and the next trial started. Six training trials were conducted changing the location of the open side of the box in-between trials (left, right, back) before proceeding to the test phase.

In the test phase, the box was uncovered and animals were no longer allowed to witness the baiting process. Accordingly, the animals were put into an adjacent room during the baiting process and taken directly to the start position from there. In addition to changing the open side of the box (left, right, back) in-between trials, also the reward location was manipulated (centre = in centre of box; deep = touching the wall opposite to the open side). The experimenter was already standing motionless in the back of the baited box when the animal entered the test room again. Animals were released from the starting position and had 30 s to retrieve the reward. If this time elapsed without the animals succeeding, the trial was terminated and the animal called back to the starting position. Some animals were afraid to put their head completely inside of the box, especially in the deep reward condition; hence the experimenter helped these individuals to get the reward by pulling the lid out of the box as soon as the subject’s nose entered the box. Accordingly, we measured only the latency it took the animals to insert their nose into the box. Six test trials were conducted (open side and reward locations randomly but counterbalanced). Each combination of the open side (left, right, back) and reward location (centre, deep) was tested once.

#### Variables

The following behaviours were coded: duration close to box (with 1 m radius), latency to success (time from release until nose was inserted in box; max. 30 s), number of successful trials, and the frequency of errors (= surface touches with either nose or paw). The latter variable was considered the main measure for inhibition in this test (e.g. Lockman and Adams 2001).

### Middle cup test

**Fig. 2:**
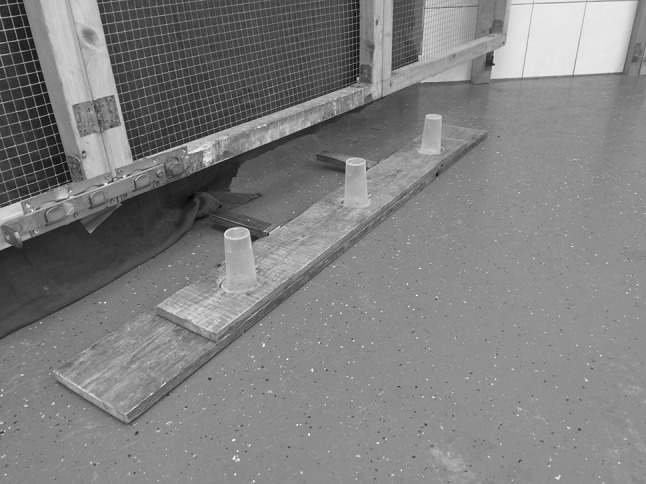
Setup for middle cup test

The middle cup tests consisted of a repeated choice between three transparent plastic cups (height: 15 cm) of which only two were baited with dry food. The cups were aligned on a wooden board (160 cm with 45 cm distance in-between cups) in notches to enhance their stability (see Fig. 2). The cups were placed in notches in the board. The board could be moved via two sticks from behind a curtain, to allow the experimenter to manipulate the cups without being visible to the animals, and additionally to stabilize the board during their choices. A fence was constructed across the corner of the test room and served as visual and physical barrier between experimenter and animals.

Since the animals were only allowed to make two choices, this test measured the motor inhibition of the animals to knock over empty cups (loss of a reward). In contrast to the box test, which also aimed at measuring motor inhibition, this test additionally involved a choice component, which was absent in the box test.

#### Procedure

We started this test with two warm-up trials, in which only one cup was visibly baited (i.e. moving the piece of dry food in front of the curtain before placing it in the notch) and placed on the board (location of cup was counterbalanced across animals). These warm-up trials were conducted to familiarize the animals with the action of knocking over cups to gain access to the reward beneath. A trainer released the subject from a starting position 2 m in front of the fence and allowed to knock over the cup and eat the reward. After the trial, the animal was called back to the starting position. If an individual had problems knocking over the cup by itself (e.g. afraid of sound), the experimenter helped, and lifted the cup once the animal had touched it with its nose. If this help was necessary, the procedure was maintained throughout testing.

In the following test phase, the cups were baited according to two conditions: experimental condition [i.e. non-adjacent cups (left and right)], and control trials [i.e. adjacent cups (left and middle, right and middle)]. The baiting procedure was the same as during the warm-up trials, only that now all three cups were visibly aligned behind the board while the experimenter showed both pieces of dry food (one in each hand) and placed them in the notches simultaneously. In the next step, the experimenter placed the cups one at a time upside-down on the notches (randomized and counterbalanced order). Once all cups were placed on their notches, and the experimenter moved her hands to the sticks, the animal was released. The individuals were allowed to make two choices, while the experimenter prevented them from knocking over the third cup by pulling the board back and putting the hand on the remaining cup. The trainer then called the animal back and the next trial started. Twenty trials were conducted with ten trials for each condition [experimental and control (five left and middle cup, five right and middle cup)] interspersed among the rest. The order of conditions was semi-randomized (i.e. no more than three trials per condition in a row) and counterbalanced.

#### Variables

In the middle cup test, we coded the following variables: frequency of correct choices (= both choices correct), latency to make both choices (= time from release until second choice was made), and the time spent in proximity to cups (= within 1 m of board). The main measure for inhibition in this test is considered to be the ratio between correct choices in the control condition and the experimental condition (e.g. Amici et al. 2008).

### Reversal learning test

**Fig. 3:**
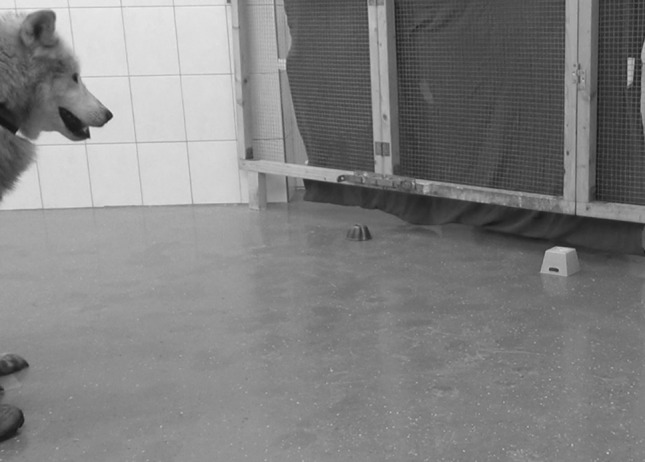
Objects used for reversal learning test arranged in front of the curtain at the beginning of a trial. The wolf is held at the starting position

In a first step the animals learned to discriminate between two objects (green quadratic-shaped plastic object (11.5 × 11.5 cm) and blue metal cake tin (16 cm diameter); see Fig. 3) and to form an association between the choice of a specific object and a reward (S+; piece of sausage). The objects were baited from behind a covered wire fence that was positioned across the corner of the test room, and served as a barrier between experimenter and animals. In a second step, these object contingencies were reversed in a way that the previously negative object (S−) was now being rewarded.

Accordingly, this test aimed at measuring cognitive inhibition by assessing the animals’ capacity to inhibit choosing S+ in the reversal phase.

#### Procedure

This test was preceded by a warm-up phase in which the animals were presented with only the positive object (S+), to facilitate the learning process. For half of the subjects, the green plastic object was set as S+, whereas for the other half the blue cake tin was set as S+. The assignment to either group was random but counterbalanced across subjects. The experimenter showed the subject the piece of reward (sausage) by lifting the hand with the reward in front of the curtain before placing it on the ground and turning the positive objects on top of it. As soon as the experimenter withdrew her hand behind the curtain, the animal was released from a starting position 2 m in front of the fence. Touching the object with the nose was considered a choice and along with a clicker sound the experimenter lifted the object. The animal was allowed to eat the reward and then called back by the trainer. Four warm-up trials were conducted.

The following test phase was divided into two phases: acquisition and reversal phase. The procedure was identical to the warm-up phase, except that baiting was invisible to the subject (i.e. baiting behind the curtain), and both objects were pushed in front of the curtain simultaneously. The subject was released from the starting position and allowed to make one choice. A choice was noted when the subject touched an object with its nose. If this was the correct object (S+) a click sound was emitted and the object lifted by the experimenter. However, if the choice was incorrect (S−), no sound was emitted and the empty object was lifted. In addition, the S + object was quickly lifted without giving the subject access to the reward, to give the animals the chance to see where the reward was hidden. The trainer then called the individual back and the next trial began. Twelve trials were conducted per session with semi-randomized but counterbalanced locations of the S+ object (i.e. not more than twice on the same side). A subject reached learning criterion when it chose S+ in at least 9 out of the 12 trials (binomial: *p* = 0.02). If this criterion was not met within one session, another session was conducted after a short break (2–3 min), in which the animal left the test room. No more than three sessions were performed per test day. All individuals reached learning criterion within four test sessions. Once the animals reached this criterion, the reversal phase started after a short break. Specifically, in the reversal phase, the object contingencies were reversed (i.e. S+ no longer rewarded, S− rewarded). This phase started again with four warm-up trials (only S−), however, from now on the clicker was omitted. The test trials were performed as before, but no learning criterion was set. Only one test session with 12 trials was conducted in the reversal phase.

#### Variables

We coded the following variables: frequency of correct choices (i.e. S+ in last successful acquisition phase, S− in reversal phase), latency to make a choice (time from release to choice), duration close to objects before making a choice (within 1 m radius). The ratio between correct choices in the last acquisition phase (in which the learning criterion was reached) and the reversal phase was considered the main measure for inhibitory control in this test.

### Buzzer test

**Fig. 4:**
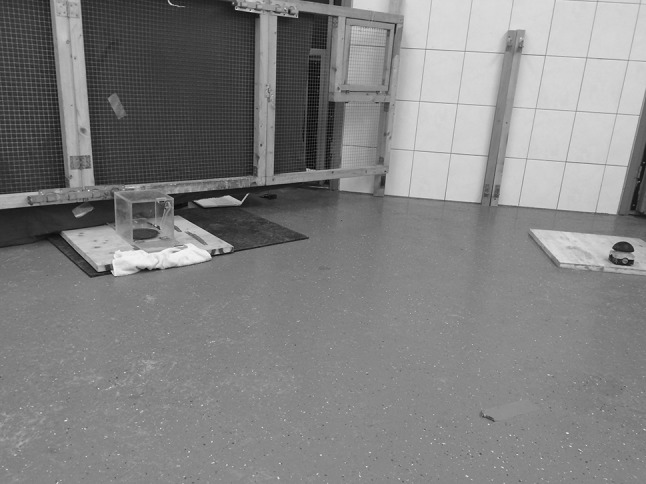
Setup for buzzer test. The transparent box is located in front of a curtain with an experimenter opening the box from behind the curtain. The buzzer is located at a distance of 2 m

In this test, the animals were confronted with a transparent box (25 × 25 × 25 cm) that contained a piece of sausage and which could be opened by pressing a buzzer (Eaton^®^ FAK-S/KC11/I) located away from the box (side counterbalanced across subjects). The front side of the box could be opened via an opening mechanism (string attached to a latch) from the opposite side of the box (see Fig. 4). The box was positioned in front of a fence covered with a curtain, which served as a visual and physical barrier for a second experimenter that was responsible for opening the box by pulling the string, as soon as the buzzer was pressed. Both box and buzzer were screwed to separate wooden boards to enhance stability.

Accordingly, to gain access to the reward, the individuals needed to inhibit manipulating the box but instead turn away from the reward and press the buzzer. This test included components of motor inhibition (i.e. manipulating the box, movement towards the buzzer), but also aspects of cognitive inhibition (i.e. referring to learned procedure (= pressing buzzer) in a tempting situation).

#### Procedure

In a pre-training phase, the animals were trained to press the buzzer. Since the animals already knew how to press a buzzer from previous studies (e.g. Essler et al. 2017), this behaviour was only refreshed in several trials (5–7 trials). In doing so, the experimenter was standing next to the buzzer and pointed towards it, while rewarding the animal (i.e. food and clicker) once it pressed the buzzer. After re-establishing this behaviour, the experimenter opened and closed the opaque box several times to familiarize the animal with the opening sound of the box. Additionally, after having opened the box several times, a food reward was placed inside the box and the animal was allowed to retrieve the reward once the experimenter had opened it. Only when the animal showed no fear of the opening sound and willingly retrieved the food out of the box, the training trials began.

In the training trials, the experimenter visibly baited the opaque box by placing a piece of sausage on a blue plastic lid inside the box and closed the box. The buzzer was positioned next to the box (50 cm distance) on either the left or right side (counterbalanced across subjects), and the animal was held on a collar by a trainer 2 m away from the box. After baiting and closing the box, the experimenter moved several steps away and the subject was released. If the animal pressed the buzzer, a click sound from the clicker was emitted, the box was opened (string pulled by helper behind the curtain), and the subject was allowed to eat the reward. If the animal did not press the buzzer within 10 s, the experimenter helped by approaching and pointing towards the buzzer. After each trial, the trainer called back the subject and the next trial started. The experimenter moved further away from the buzzer after each successful trial until she was standing 3 m away from it. Training criterion was met when an animal pressed the buzzer without help from the experimenter in seven consecutive trials (dogs: 8.75 ± 3.73 training trials; wolves: 8.67 ± 4.14 training trials).

For the test trials, the box was uncovered and the buzzer was moved to a position 2 m away from the box on either the left or right side (same side as in training trials). As before, the experimenter visibly baited the box while the trainer held the animal by the collar 2 m away from the box. After closing the box, the experimenter walked towards the buzzer, pressed it and then moved on to a position behind the subject’s back. When the experimenter reached this position the animal was released and had 60 s to press the buzzer. If the buzzer was pressed, the box was opened and the animal was allowed to eat the reward. If the subject did not press the buzzer within 60 s, the trial was terminated and the animal called back before the next trial started. Depending on the animal’s success in the first trial, in the next trial the pressing of the buzzer was omitted and the experimenter only moved in the back of the animal after closing the box. If the subject was not successful, the second trial was conducted like the first one (i.e. experimenter pressing the buzzer after baiting the box). Five test trials were conducted.

#### Variables

We coded the following behaviours: duration spent in proximity to box (within 1 m radius), latency to press buzzer (time from release to pressing buzzer, max. 60 s), duration manipulating the box (scratching, licking, biting, pushing with nose), and number of successful trials (buzzer pressed within 60 s). We considered the variable ‘proximity to box’ as the main measure of inhibition (as in Brucks et al. 2017a). One dog did not complete this test since she was afraid of the box opening sound (see Supplementary Table S1).

### Analyses

Videos were coded using Solomon Coder (^**©**^András Peter, solomoncoder.com). The data were analysed using IBM SPSS and R. Following the procedure of Brucks et al. (2017a), we first analysed each test separately using non-parametric statistics (i.e. Wilcoxon tests) to validate that the test conditions were indeed more difficult than the control conditions. Moreover, a Spearman correlation was performed including the main inhibition measures for each test. Since the variables from the single tests were based on different scales, we transformed them into *z*-scores and used these scores for further analyses. In a second step, we wanted to investigate whether the inhibition measures show an underlying structure; hence we conducted principal component analyses (PCA) on each single test. A separate PCA was performed for each test including all variables coded for the specific test. We assessed sub-species differences in each test using the components provided by the single PCAs using linear models (LM). Once we established these single components, we performed an overall PCA including the single tests components. PCAs were run using Quartimax rotations and components with a bigger Eigenvalue than 1 were retained in the final solution. To compensate for missing data, we calculated individual component scores based on the results of the PCAs. Accordingly, we first *z*-transformed the raw variables, and subsequently, derived the single test components by calculating the mean of the variables loadings (based on the initial template PCA). Loadings of at least 0.5 were included and negative loadings were considered in the calculation by multiplying with − 1. No component score were calculated if more than 2/3 of the variables, which loaded on a particular component, were missing (*N* = 2). Finally, the derived single test components were used to calculate the overall PCA components (based on the overall PCA template), following the same procedure as before (i.e. mean of loadings with at least 0.5 and no more than 2/3 of component values missing). With these final inhibition components we tested whether subspecies (factor: wolf, dog), age (continuous), and sex (factor: male, female) affected the results using linear models. We used the Akaike information criterion (AIC) to find the best fitted model by reducing the full model.

A second coder, blind to the purpose of the study, coded 30% of the videos (intra-class correlation coefficients (ICC) for continuous variables: all ICC (consistency) for box test < 0.99, middle cup test < 0.91, reversal-learning test < 0.76, buzzer test < 0.98; Cohen’s kappa for frequencies: all kappa for box test < 0.65, middle cup test < 0.91, reversal-learning test < 0.94, buzzer test < 1.00).

## Results

### Correlation inhibition measures

The main measures from each inhibition test were not correlated with each other, neither for the dogs nor for the wolves (all *r*_s_ < 0.37; see Table 1).

**Table 1:** Spearman correlation matrix between z-transformed inhibition measures from each test separated for dogs (*N* = 16) and wolves (*N* = 12)

Subspecies	Test			
	Box	Middle cup	Reversal learning	Buzzer
	Errors	Ratio control/exp	Ratio aqu./rev	Vicinity
Dogs				
Box		*r*_s_ = − 0.17	*r*_s_ = 0.11	*r*_s_ = 0.37
MC			*r*_s_ = 0.21	*r*_s_ = − 0.08
RL				*r*_s_ = − 0.08
BUZ				
Wolves				
Box		*r*_s_ = − 0.24	*r*_s_ = 0.42	*r*_s_ = 0.15
MC			*r*_s_ = 0.12	*r*_s_ = 0.00
RL				*r*_s_ = 0.31
BUZ				

*BUZ* buzzer test, *MC* middle cup test, *RL* reversal learning test

### Box test

The animals spent more time in vicinity to the box when it was transparent compared to the training phase with an opaque box (Wilcoxon test: *T* = 393, *N* = 28, *p* < 0.001). Moreover, they committed more errors i.e. scratching the wall when the box was transparent than when it was covered (Wilcoxon test: *T* = 339.5, *N* = 28, *p* < 0.001). Interestingly, the reward location (deep or centre location) did not affect the latency to retrieve the reward (Wilcoxon test: *T* = 248.5, *N* = 28, *p* = 0.156). This indicates that the visibility of the reward affected the animals’ performance, while the exact location of the reward did not. Accordingly, the variable ‘latency to success’ was not considered separately for each reward location (contrary to Brucks et al. 2017a, b) but included as a sum of both trial types in the subsequent analysis.

A PCA revealed that the measures of the box test grouped on two factors (see Table 2). The latency to retrieve the reward, the time spent in proximity to the box, as well as the success in retrieving the reward grouped on one factor, which we called ‘flexibility’. Animals that needed longer to find the open side, hence spent more time close to the box, and were less successful, lacked explorative abilities but also the flexibility to try out other options to solve this test. The second component included the number of surface touches with either nose or paw and was labelled ‘perseveration’. Animals scoring high on this component committed more errors and hence displayed more perseverative behaviours. These two components explained 84.7% of variance in the data. The dogs’ performance in this test did not differ from wolves (flexibility component: *F* = 1.087, *p* = 0.307; perseveration component: *F* = 0.359, *p* = 0.555).

**Table 2:** Principal component analysis for variables of box test

	Components	
	Flexibility	Perseveration
Latency to success	0.98	
Success	− 0.94	
Time vicinity to box	0.74	
Nose errors		0.91
Paw errors		0.86
% variance	50.02	34.72
Eigenvalue	2.50	1.74
Cronbach’s *α*	0.88	0.75

KMO = 0.5; Bartlett: *χ*^2^_10_ = 97.0, *p* < 0.001

### Middle cup test

More successful choices were made in the control condition (5.45 ± 1.41; adjacent cups baited) compared to the experimental condition (0.79 ± 1.05; non-adjacent cups baited; Wilcoxon Test: *T* = 435, *N* = 29, *p* < 0.001). The latency to make choices did not differ between test conditions (Wilcoxon test: *T* = 195.5, *N* = 29, *p* = 0.382). Accordingly, this variable was included as a sum of both trial types (contrary to Brucks et al. 2017a, b).

The variables from this test grouped on two components (see Table 3). The latency to make choices, duration spent close to cups, as well as the number of correct choices in the experimental trials grouped together on one component. We labelled this component ‘decision time’ since the majority of the variables measure the animals’ motivation and speed in making their choices. The grouping of the variable ‘correct choices in experimental trials’ is rather surprising, considering its rather low loading of 0.58, as well as the limited success of individuals in these trials. Accordingly, we neglected it in the interpretation of this component. The second component was solely comprised of the variable correct choices in control trials. Considering that animals needed to pay attention to the baiting side of the rewards, we labelled this component ‘attention’. These two components explained 75.3% of the variance observed in the data. The dogs exhibited a slower decision time than the wolves (LM: *β* = − 0.89, *SE* = 0.34, *t* = − 2.38, *p* = 0.015), while we found no difference between dogs and wolves in the attention component (*F* = 0.126, *p* = 0.725).

**Table 3:** Principal component analysis for variables of middle cup test

	Components	
	Decision time	Attention
Latency to choice	0.91	
Duration close to cups	0.85	
Correct choices exp. trials	0.58	
Correct choices control trials		0.93
% variance	48.43	26.90
Eigenvalue	1.94	1.08
Cronbach’s *α*	0.88	NA

KMO = 0.6; Bartlett: *χ*^2^_6_ = 15.6, *p* = 0.016

### Reversal learning test

The animals needed 1.45 ± 0.87 sessions to reach the training criterion. Significantly less correct choices were made in the reversal phase (4.24 ± 2.52 correct choices) compared to the last acquisition phase, in which the criterion was reached (10.01 ± 1.01 correct choices; Wilcoxon Test: *T* = 0, *N* = 29, *p* < 0.001). Furthermore, they took more time to make a choice in the reversal phase compared to the acquisition phase (Wilcoxon test: *T* = 9, *N* = 29, *p* < 0.001).

The PCA revealed that the measured variables grouped on two components (see Table 4). The first component included measurements of the duration spent close to the objects, and the latencies to make a choice in the last acquisition phase as well as in the reversal phase. We labelled this component ‘choice time’ since the variables loading on this component are all measures of the individuals’ decision time. The second component was comprised of the variables ‘sessions needed to reach the criterion’ and the number of correct choices in the reversal phase. Since these two variables measure the animals’ ability to learn new associations, we labelled this component ‘flexibility’. These components explained 74.4% of the variance in the data. We found that dogs exhibited a longer choice time compared to wolves (LM: *β* = − 0.75, SE = 0.32, *t* = − 2.63, *p* = 0.026), while we found no difference between dogs and wolves in the flexibility component (*F* = 0.051, *p* = 0.823).

**Table 4:** Principal component analysis for variables of reversal learning test

	Components	
	Choice time	Flexibility
Duration close to objects	0.96	
Latency acquisition phase	0.89	
Latency reversal phase	0.89	
Sessions to criterion		0.82
Correct choices reversal		− 0.75
% variance	52.89	21.54
Eigenvalue	2.67	1.23
Cronbach’s *α*	0.91	0.33

KMO = 0.7; Bartlett: *χ*^2^_10_ = 55.4, *p* < 0.001

### Buzzer test

The animals learned the association between buzzer and box within 8.7 ± 3.8 trials. They manipulated the box for a longer time in the test phase, when it was transparent, compared to the opaque version in the training phase (Wilcoxon test: *T* = 310, *N* = 28, *p* < 0.001). Moreover, the latency to press the buzzer was significantly longer in the test phase compared to the training phase (Wilcoxon test: *T* = 24, *N* = 28, *p* < 0.001). This indicates that the animals were more inclined to stay close to the box when they could see the reward inside, and additionally needed longer to press the buzzer confirming that the test condition was indeed more difficult for the animals.

The measurements of this test grouped on two components (see Table 5). The latency to press the buzzer as well as the overall success grouped together on one component. We labelled this component ‘inactivity’ since it captures variables related to the animals’ motivation and activity. The second component included measurements for the manipulation of the box and the time spent in proximity to the box, hence we labelled this component ‘persistency’ since those variables measure the general involvement and endurance in getting access to the reward. These two components explained 93.8% of the variance within the data. The dogs did not differ from the wolves in the test components (inactivity: *F* = 0.418, *p* = 0.524; persistence: *F* = 0.363, *p* = 0.553).

**Table 5:** Principal component analysis for variables of buzzer test

	Components	
	Inactivity	Persistence
Success	− 0.98	
Latency press buzzer	0.97	
Manipulate box		0.95
Vicinity to box		0.85
% variance	58.10	35.72
Eigenvalue	2.32	1.43
Cronbach’s *α*	0.96	0.81

KMO = 0.5; Bartlett: *χ*^2^_6_ = 91.5, *p* < 0.001

### Overall PCA

The overall PCA included the components derived from the single tests and revealed three underlying components, which explained 71.7% of the variation within the data (see Table 6). Factors related to the animals’ decision speed and attention loaded on the first component, in particular, the ‘choice time’ factor from the reversal learning test, the ‘decision time’ and ‘attention’ factor from the middle cup test, and the ‘inactivity’ factor from the buzzer test. Accordingly, we labelled this component ‘motivation’ since it captured the individuals’ activity and speed in making a choice, but also their attention. The second component included the ‘flexibility’ components from the box and reversal learning tests, as well as the persistency component from the buzzer test. Considering that these components all measure the animals’ adaptability to changing aspects of the test (e.g. open sides, object contingencies), but also their persistence in sticking with their initially learned behaviour, we labelled this component ‘flexibility’. The final component included the ‘perseveration’ factor from the box test and the ‘persistence’ factor from the buzzer test, accordingly, we labelled this component ‘perseveration’.

**Table 6:** Overall principal component analysis

	Components		
	Motivation	Flexibility	Perseveration
RL: choice time	0.80		
MC: decision time	0.76		
BUZ: inactivity	0.71		
MC: attention	− 0.63		
Box: flexibility		0.78	
RL: flexibility		0.71	
Box: perseveration			0.91
BUZ: persistence		− 0.52	0.75
% variance	32.75	20.41	18.68
Eigenvalue	2.62	1.63	1.49
Cronbach’s *α*	0.71	0.42	0.59

KMO = 0.5; Bartlett: *χ*^2^_28_ = 47.6, *p* = 0.01

### Dog–wolf differences

To detect whether the dogs and wolves differed in their inhibitory control abilities, we ran linear models on the inhibition components derived from the overall PCA. We found an age—subspecies interaction for the ‘motivation’ component (LM: *β* = 0.24, SE = 0.11, *t* = 2.11, *p* = 0.045). Accordingly, younger dogs scored higher on this component than older dogs, whereas no such effect could be found for the wolves. Thus indicating that younger dogs are less motivated than older dogs or wolves. For the other two components no effects emerged (see Table 7).

**Table 7:** Summary of linear model outputs for effects of subspecies, age, and sex on inhibition components

Component	Subspecies	Age	Sex
Motivation	*F* = 4.47		*F* = 0.18
	*p* = 0.045*		*p* = 0.678
Flexibility	*F* = 0.76	*F* = 0.74	*F* = 1.37
	*p* = 0.266	*p* = 0.398	*p* = 0.235
Perseveration	*F* = 0.30	*F* = 0.23	*F* = 0.37
	*p* = 0.590	*p* = 0.637	*p* = 0.548

*p < 0.05

## Discussion

As in previous research, we found that each task taken separately did appear to measure a different aspect of inhibitory control since the measures from the different tests did not correlate with one another. However, we found three components, which are likely linked to behavioural inhibition. Finally, we could reveal that these inhibition components did not differ between dogs and wolves.

When analysing the tests separately, we found that each test captured the animals’ inhibitory control abilities in line with previous studies. The control/training conditions were consistently easier to solve than the test conditions. Interestingly, however, some variables, which showed a difference between test and training in our previous study with pet dogs (Brucks et al. 2017a), did not differ in the current study. In the box test, the location of the reward (deep or centre) did not affect the latency to retrieve the reward. This is in contrast to other studies, in which animals had more difficulties in finding the open side of the box, when the reward was placed deeply inside (Brucks et al. 2017a; Lakshminaryanan and Santos 2009; Santos et al. 1999). Moreover, in the middle cup task, no differences emerged in the latency to make choices between the control and experimental condition (median control: 83.0 s, experimental: 79.2 s). Contrary to this, the pet dogs in our previous study took more time to make their choices in the experimental condition (median control: 60 s, experimental: 66 s; Brucks et al. 2017a). Additionally, the animals in the current study needed overall longer to make their choices. In particular, the pack dogs were slower to choose cups compared to the pet dogs (median pack dogs: control: 107 s, experimental: 89 s). Moreover, the dogs and wolves performed very poorly in the middle cup test in general. On average only half of the control trials, which in theory should not be taxing, resulted in a gain of both rewards (median dogs: 5.0, wolves: 5.5 correct choices), which is slightly worse than the performance of the pet dogs in our previous study (median: 6 correct choices; Brucks et al. 2017a). However, the dogs and wolves performed significantly worse in the test than the control condition (median: pack dogs: 1, wolves: 0 correct choices) with only little individual variation (range pack dogs: 0–3, wolves: 0–2 correct choices). Likewise, the pet dogs in our previous study showed a poor performance on a group level (median: 1 correct choice; Brucks et al. 2017a); however, they exhibited more individual variation with some individuals choosing correctly in every single trial of the test condition (range pet dogs: 0–10 correct choices). This rather bad performance in the test condition confirms that the middle cup test was indeed eliciting inhibitory control problems. The observed differences in performance between the pack dogs and the pet dogs from the previous study (Brucks et al. 2017a) indicate that the rearing and living environment (outdoor enclosure vs. family home), social environment (pack-structure vs. human household), but also the training experience (standardized training and cognitive testing vs. individual training) might have affected the dogs’ inhibition abilities required for this specific test. Indeed, the effect of experience and motivation on the behaviour in question is often neglected in comparative studies (see Rowe and Healy 2014 for a review). While these effects are controlled for in the wolf–dog comparison due to same living/rearing conditions, and similar feeding regime at the WSC (see Range and Virányi 2013 for a detailed description of rearing and living conditions), a direct extension of the results to other species, including pet dogs, is potentially difficult. Controlling for these effects of socialisation (i.e. training and experience) on complex behaviours, such as inhibitory control, is a major requirement in gaining truly comparative data.

Despite the fact that the analyses confirmed that each tests measured the animals’ inhibitory control abilities, the performances in the single tests were not correlated with each other—neither in dogs nor wolves. This finding further supports the results of previous studies suggesting that inhibitory control is indeed context-specific (e.g. Bray et al. 2013; Brucks et al. 2017a), and justifies our test battery approach to assess species-specific inhibition capacities.

In an attempt to understand whether the animals’ behaviour was consistent across tests, we utilized an exploratory approach. And indeed, three components emerged, which seemed to capture the underlying structure of the animals’ behaviour. We labelled those inhibition components ‘motivation’, ‘flexibility’ and ‘perseveration’. The motivation component included variables describing the general motivation to get access to food rewards and pay attention to the reward location. The motivation to explore novel situations plays an important role in problem-solving success (e.g. Benson-Amram and Holekamp 2012; Thornton and Samson 2012). If an individual is hindered in exploring situations due to neophobia or a lack of food motivation it will likely not engage in problem-solving. The fact that variables related to the individuals’ motivation loaded on a separate component in this study further highlights the importance of considering individual differences in motivation when assessing cognitive abilities, such as problem-solving skills (see discussion above, and also Rowe and Healy 2014). Another aspect that emerged in our analysis is the ability to flexibly switch between options, an aspect which has often been linked to success in changing environments (e.g. Bond et al. 2007). Measures related to this ability grouped on our second component, which we labelled flexibility. Variables loading on this component relate to the individuals’ ability to adjust their behaviour to changing situations (i.e. reversed object contingencies in reversal-learning test, visibility of rewards in transparent instead of opaque boxes in buzzer and box test). And indeed, to flexibly adapt to novel situations an individual has to first inhibit previously learned response patterns. Finally, our last component was labelled perseveration and included measures related to preservative behaviours during the tests. Perseverative actions (i.e. repetition of same behaviours independent of feedback) are thought to occur due to inhibition problems and can prevent individuals from flexibly adjusting to a given situation (see Hauser 1999 for a review). These three inhibition components were rather similar to our previous study with pet dogs (Brucks et al. 2017a) in that the same variables grouped together, even though one test was not included in the present study. Accordingly, these results strengthen the validity and robustness of our previous test battery in assessing inhibitory control abilities. This test battery is thus very well suited to test inhibitory control abilities in canines and could potentially be adopted for other species as well, to gain a better understanding of how different aspects of inhibitory control influence problems solving skills and other complex behaviours.

Finally, using these inhibition components, we investigated whether dogs and wolves differ in their inhibitory control abilities. We found an effect only in the motivation component, whereas the flexibility and perseveration component did not show any differences between dogs and wolves. Specifically, dogs scored higher than wolves on the motivation component, which translates into a lower motivation in dogs (i.e. time to retrieve the reward and follow reward placements). In particular, the dogs’ motivation was characterized by an age effect and younger dogs showed lower motivation than younger wolves. This age effect, however, needs to be treated with caution since the age distribution was not well balanced. In particular, no younger wolves were tested, making it difficult to assess the robustness of this result. Furthermore, an increased sample size would have allowed for statistically more robust interpretations, however, since keeping and raising wolves and dogs in a similar environment requires specific facilities and extensive care, sample sizes are always constraint for investigating these questions. Accordingly, it needs to be noted that the results of the PCAs need to be treated carefully due to the low sample size (i.e. KMO as measure for sampling adequacy on the border of being acceptable; see Budaev 2010). Nonetheless, the present study did confirm our previously found inhibition components in pet dogs (Brucks et al. 2017a), which was based on a bigger sample size. Still, the fact that the dogs and wolves did not differ in the majority of inhibition components indicates that domestication might not have affected inhibitory control *per se*. According to the domestication hypotheses (Gácsi et al. 2009; Hare et al. 2005, 2012), dogs are expected to show enhanced inhibition skills due to the selection of certain traits (i.e. fearlessness, reduced aggression, inhibited temperament) during the course of domestication. However, our data do not support these hypotheses since dogs’ inhibition abilities were not different from the wolves’ abilities. Nonetheless, as previous research has shown socio-ecological factors can account for species differences in inhibitory control abilities as well (e.g. Amici et al. 2008; MacLean et al. 2014; Stevens et al. 2005). Since dogs show a lower social complexity than wolves (i.e. no cooperative breeding and hunting) and a feeding ecology that requires less inhibitory skills (i.e. stable resources), it would follow that dogs should show decreased inhibitory control abilities compared to wolves. However, this was not the case. This result might be seen as support for the claim that inhibitory control is strongly context-specific. While wolves might require enhanced inhibition skills in the context of ecological and social decisions, dogs potentially do not require such skills. Reversed predictions could be made in regard to social interactions with humans, as dogs seem to exceed in understanding human gestures, which also require inhibition (e.g. looking at humans and not at reward locations), wolves do seem to have inhibition problems in this context and cannot divert their attention away from the reward location to the human (e.g. Gácsi et al. 2009, but see; Udell et al. 2008).

The inconclusive results of previous studies about dogs’ and wolves’ inhibition abilities can potentially be explained by the context-specificity of inhibitory control as well. For example, Marshall-Pescini et al. (2015) found that dogs outperformed wolves in one task, but not in another. It is very likely that the two inhibition tasks (cylinder and fence-detour) did not measure the same aspects of inhibitory control. To gain a more complete assessment of species-specific inhibitory control capacities it is essential to use multiple inhibition tests, which aim at measuring different aspects of inhibitory control, as was done in the current study. Moreover, the differences in dogs’ and wolves’ abilities to focus and cooperate in choice paradigms (Gácsi et al. 2009), but also to solve problems without the help of humans (Udell 2015) might measure yet another aspect of inhibitory control, namely social inhibition. Dogs are potentially more inhibited in the presence of humans than wolves, which is possibly mirrored in their increased gazing behaviour towards the human and lower frequency to struggle when contained by the experimenter in these experiments (Gácsi et al. 2009; Miklósi et al. 2003; Udell 2015). Indeed, dogs might exhibit differential inhibition skills depending on the social partner (conspecific vs. human). Accordingly, wolves seem to excel at tasks that require inhibition in an intraspecific cooperative context (i.e. coordinating and waiting for actions of conspecific partner), which is in line with the predictions derived from the socio-ecological background, while dogs failed to coordinate with each other (Marshall-Pescini et al. 2017b). Moreover, it has recently been shown that dogs and wolves differ in their tolerance towards conspecifics (e.g. Range et al. 2015). Wolves generally exhibit less often sever aggressive behaviours towards pack members than dogs (Cafazzo et al. 2018), hence dominant wolves might be better in inhibiting their aggressive behaviours towards lower ranking individuals than dogs, which in turn also facilitates cooperation. Potentially, social and non-social inhibition are separate constructs, while additionally, domesticated species might show different social inhibition towards conspecific and human partners. In the present study, we focused on conducting non-social inhibition tests in a limited social context (i.e. no direct interactions with humans, although humans were present during the test), but future studies need to assess whether dogs and wolves differ in their social inhibition abilities (with conspecifics and human partners; e.g. as in Leonardi et al. 2012; Reddy et al. 2015). Furthermore, inconclusive results from previous studies might be attributed to other aspects such as training, experiences, and the living environment, which can affect inhibitory control abilities. Especially tests involving a learning component such as the reversal-learning test or buzzer test are potentially easier to learn for animals that are familiar with the general features of cognitive tasks (e.g. discrimination, shaping procedure, etc.). But also experiences with certain characteristics of inhibition tasks (i.e. transparent surfaces, fences) could influence the animals’ performance in these tests. In addition to a genetic component, experiences during ontogenesis could play an important factor in acquiring inhibitory control. For example, highly socialized wolves can outperform dogs in understanding human social cues (Udell et al. 2008), while unsocialized shelter dogs seem to have problems in following human gestures (e.g. D’Aniello et al. 2017). Similarly, as discussed above, ontogeny seems to play some part in the acquisition of inhibitory control, since pet dogs seem to outperform pack dogs in theses inhibition tasks. Accordingly, in addition to the genetic component also an ontogenetic component seems to play an important role in shaping dogs’ behaviour (e.g. Udell and Wynne 2010). The relative weight of these components, however, remains unknown; thus wolves might acquire similar levels of inhibitory control if extensively socialized to humans, although the feasibility and ethical considerations of raising wolves in a human environment are limiting factors in answering this question. Consequently, it remains of major importance to control for these effects when testing animals with different social backgrounds (e.g. pet dogs, shelter dogs, socialized wolves).

In conclusion, we could show that dogs and wolves exhibit equal levels of inhibition abilities as assessed in a non-social test battery. Moreover, in line with previous studies, the individual’s performance across tests was not correlated, but inhibition components could be extracted that explained the individual variation across tests (i.e. motivation, flexibility and perseveration). The results of the current study can be explained by various evolutionary scenarios and thus raise multiple questions in regard to the selection processes that might have affected inhibitory control capacities during the course of domestication.

## Electronic supplementary material

Below is the link to the electronic supplementary material.

Supplementary material 1 (DOCX 59 KB)

Supplementary material 2 (XLSX 49 KB)
