# Supplementary material for: Dogs and wolves do not differ in their inhibitory control abilities in a non-social test battery
Source: Anim Cogn. 2018 Oct 3;22(1):1–15. doi: 10.1007/s10071-018-1216-9 (PMC6326967; doi:10.1007/s10071-018-1216-9)
Supplement: Supplementary file 1 — Supplementary material 1 (DOCX 59 KB) [file 10071_2018_1216_MOESM1_ESM.docx]

**Supplementary Material**

**Table S1.** Individual characteristics of dogs and wolves tested in the inhibition tests.

| **Name** | **Species** | **Age (yrs.)** | **Sex** | **Missing test** |
| --- | --- | --- | --- | --- |
| Amarok | wolf | 4.6 | M | --- |
| Aragorn | wolf | 8.5 | M | --- |
| Chitto | wolf | 4.9 | M | --- |
| Geronimo | wolf | 7.5 | M | --- |
| Kaspar | wolf | 8.6 | M | --- |
| Kenai | wolf | 6.7 | M | --- |
| Nanuk | wolf | 7.5 | M | --- |
| Shima | wolf | 8.6 | F | --- |
| Tala | wolf | 4.7 | F | --- |
| Una | wolf | 4.9 | F | --- |
| Wamblee | wolf | 4.6 | M | --- |
| Yukon | wolf | 7.6 | F | --- |
| Asali | dog | 6.3 | M | --- |
| Bansai | dog | 2.8 | M | --- |
| Binti | dog | 6.3 | F | --- |
| Bora | dog | 5.5 | F | --- |
| Enzi | dog | 2.8 | M | --- |
| Gombo | dog | 2.8 | M | --- |
| Hiari | dog | 2.8 | M | --- |
| Imara | dog | 2.8 | F | --- |
| Layla | dog | 5.5 | F | Buzzer |
| Maisha | dog | 6.1 | M | --- |
| Meru | dog | 6.3 | M | --- |
| Nia | dog | 5.5 | F | --- |
| Nuru | dog | 5.6 | M | --- |
| Panya | dog | 2.8 | F | Box |
| Pepeo | dog | 2.8 | M | --- |
| Sahibu | dog | 2.9 | M | --- |
| Zuri | dog | 5.6 | F | --- |
